# Recent Investigations on the Use of Copper Complexes as Molecular Materials for Dye-Sensitized Solar Cells

**DOI:** 10.3390/molecules29010006

**Published:** 2023-12-19

**Authors:** Francesco Fagnani, Alessia Colombo, Claudia Dragonetti, Dominique Roberto

**Affiliations:** Department of Chemistry, University of Milan, UdR-INSTM of Milan, Via C. Golgi 19, I-20133 Milan, Italy; alessia.colombo@unimi.it (A.C.); claudia.dragonetti@unimi.it (C.D.); dominique.roberto@unimi.it (D.R.)

**Keywords:** dye-sensitized solar cells, photosensitizers, electron shuttles, copper complexes

## Abstract

Three decades ago, dye-sensitized solar cells (DSSCs) emerged as a route for harnessing the sun’s energy and converting it into electricity. Since then, an impressive amount of work has been devoted to improving the global photovoltaic efficiency of DSSCs, trying to optimize all components of the device. Up to now, the best efficiencies have usually been reached with ruthenium(II) photosensitizers, even if in the last few years many classes of organic compounds have shown record efficiencies. However, the future of DSSCs is stringently connected to the research and development of cheaper materials; in particular, the replacement of rare metals with abundant ones is an important topic in view of the long-term sustainability of DSSCs intended to replace the consolidated fossil-based technology. In this context, copper is a valid candidate, being both an alternative to ruthenium in the fabrication of photosensitizers and a material able to replace the common triiodide/iodide redox couple. Thus, recently, some research papers have confirmed the great potential of copper(I) coordination complexes as a cheap and convenient alternative to ruthenium dyes. Similarly, the use of copper compounds as electron transfer mediators for DSSCs can be an excellent way to solve the problems related to the more common I_3_^−^/I^−^ redox couple. The goal of this mini-review is to report on the latest research devoted to the use of versatile copper complexes as photosensitizers and electron shuttles in DSSCs. The coverage, from 2022 up to now, illustrates the most recent studies on dye-sensitized solar cells based on copper complexes as molecular materials.

## 1. Introduction

Dye-sensitized solar cells (DSSCs) are nowadays among the most studied types of solar cells; they appeared in 1991 thanks to the work of O’Regan and Grätzel [1], and since then they have received increasing attention [2]. The working principle of such devices is based on the sensitization of the electrodes through an organic compound or a coordination complex as dye: the sensitizer is characterized by proper functional groups (for example, carboxylic and/or phosphonic acid) allowing the anchoring on the semiconductor surface (usually titanium dioxide, TiO_2_) that is deposited on the conductive glass, which constitutes the anode of the cell. 

When photons having the suitable energy hit the dye, it is excited to a higher electronic state and provides an electron to the titania; then, the electron reaches the anode and, by means of an external circuit, the counterelectrode of the system. Once it reaches the cathode, the electron participates in a redox cycle with a couple of redox mediators and finally recombines with the cationic form of the dye, regenerating it in its neutral form and providing the possibility of starting a new cycle. 

**Figure 1:**
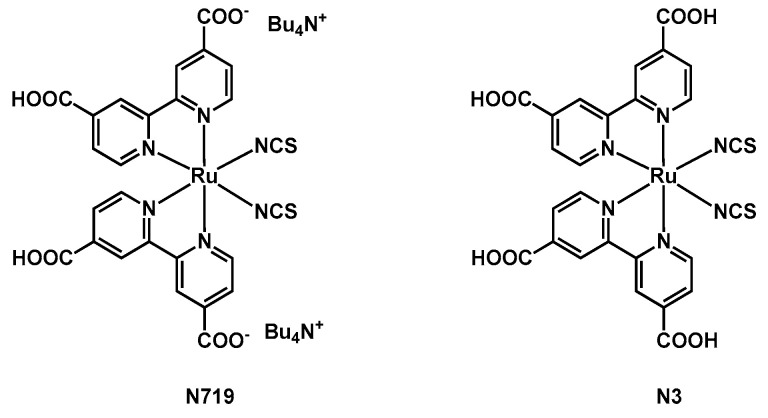
Structure of reference dyes **N719** and **N3**.

Up to now, the traditional most performing dyes involved in DSSCs are the Ru(II) complex **N3** (structure in Figure 1, having two thiocyanate ligands and two anchoring 2,2′-bipyridine-4,4′-dicarboxylic acids) and the related **N719**, in which two of the four COOH groups are presented as tetrabutylammonium salts. The problem arising from the use of these dyes is that ruthenium is a rare and expensive metal, while the presence of the labile NCS ligands can lead to the degradation of the complex via their substitution with other species present in the formulation injected into the cell [3,4,5].

Nevertheless, in the last decade, new purely organic or organometallic compounds have been applied as sensitizers in DSSCs, showing record efficiencies exceeding 13% [6,7,8,9].

Moving to the redox mediators, the most employed species are the I^−^/I_3_^−^ couple, which has some disadvantages such as: (i) I_2_ in equilibrium with I_3_^−^ is volatile, complicating long-term cell sealing even if it is tightly sealed; (ii) I_3_^−^ is darkly colored, thus limiting the light harvesting efficiency of the sensitizer; (iii) I_3_^−^/I^−^ is corrosive and will corrode most metals, posing a serious problem for the use of metal grid collectors necessary for scaling up the solar cells to large areas [10].

To overcome the mentioned problems, many possible solutions have been proposed to replace both the Ru-based dyes and the iodine-containing electron shuttles, even if, up to now, it has not been possible to reach photovoltaic performances better than those obtained by using such species.

One approach is to replace the Ru(II) dyes with complexes based on cheaper metals such as cobalt and copper; moreover, couples of Cu complexes have also been applied as redox mediators [11,12].

This review aims to illustrate the research carried out in the last two years regarding the use of copper complexes as molecular materials for dye-sensitized solar cells as an important update on some recent excellent reviews [13,14,15,16,17]. In particular, it shows how the cosensitization strategy is a fruitful way of boosting the photoconversion efficiency of copper-based DSSCs without the need for complicated organic structure design; it also reports on the birth, in 2023, of a very appealing novel strategy for efficient sensitizers consisting of homoleptic complexes of the [(DπA)_2_Cu]^+^ type, with both electron-withdrawing (A) and electron-donating (D) moieties on the same ligand. It also highlights the importance of molecular engineering of photosensitizers toward highly efficient DSSCs with copper electron shuttles, reporting on a DSSC developed in 2023 and sensitized with a well-designed organic compound that achieved an efficiency of 13.2%, a record efficiency for DSSCs based on copper electrolytes with a single sensitizer.

## 2. Copper Complexes Applied to DSSCs

In the last few years, Cu-based complexes have been proposed as key components of DSSCs, both as dyes and redox mediators. Here, novel strategies for the preparation of DSSCs based on copper complexes as molecular materials will be presented; they will be illustrated by some examples given in chronological order.

### 2.1. Copper Complexes Employed as Dyes

**Scheme 1:**
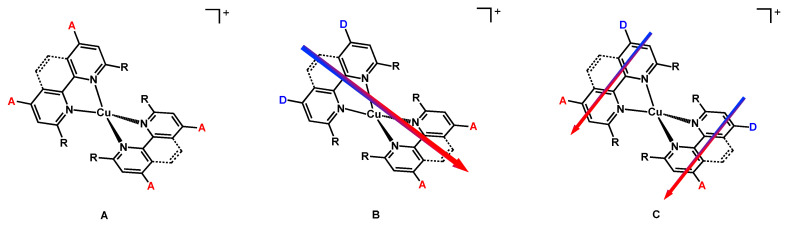
General structure of Cu(I) complexes bearing bidentate N^N ligands: compound **A** represents a generical homoleptic complex bearing electron-accepting moieties, compound **B** an heteroleptic complex based on a *push-pull* structure, while **C** the novel class of complexes presenting asymmetrical ligands, as proposed in 2023 by Housecroft et al. The electron-withdrawing substituents are marked with A, while the electron-donating ones are marked with D.

**Scheme 2:**
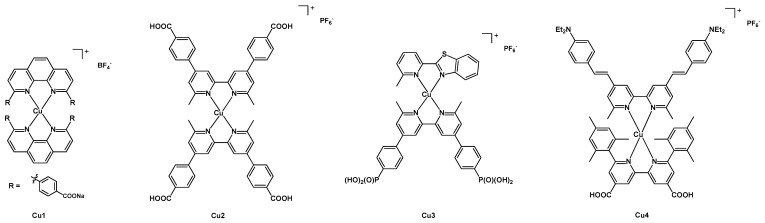
Structure of copper-based dye **Cu1**–**4**.

The first paper on the use of Cu(I) polypyridyl compounds as dyes with large band-gap semiconductors (TiO_2_ and ZnO) for DSSCs appeared in 1994 by Sauvage and coworkers [18]. By using a homoleptic copper(I) complex (see compound **A**, Scheme 1) bearing two 2,9-diphenyl-1,10-phenanthrolines substituted in *para* positions of the phenyl rings with sodium carboxylate groups (**Cu1**, Scheme 2) in DSSC with a TiO_2_ photoanode, an electrolyte based on I^−^/I_3_^−^, and a platinum counter electrode, they reached good open-circuit photovoltage (V_oc_ = 600 mV) and fill factor (FF = 0.6), but the low short-circuit photocurrent density (J_sc_ = 0.6 mA cm^−2^) led to a low efficiency (η = 0.1%). This low photocurrent could be reasonably due to a considerable steric hindrance and unfavorable electron injection into the conduction band of TiO_2_, with the presence of four deprotonated carboxylic moieties, which could also limit the anchoring to the titania surface. 

Since then, many homoleptic copper(I) complexes, mainly with chelating *N^N* ligands, have been studied in order to improve the light-harvesting properties and the ability to allow more efficient electron transfer in the conduction band of TiO_2_, leading to better photoconversion efficiency. The presence of groups such as carboxylic acids allows for good anchoring to the TiO_2_ surface. Up to 2021, to our knowledge, the best efficiency has been obtained for a homoleptic copper(I) complex bearing two 6,6′-dimethyl-2,2′- bipyridine-4,4′-dibenzoic acid ligands (**Cu2**, Scheme 2) with an efficiency of 3% (V_oc_ = 590 mV, FF = 0.69, J_sc_ = 7.3 mA cm^−2^) with unmasked cells (η_rel_ = 33% where η_rel_ is the efficiency relative to **N719** set at 100%; for a better comparison between the results of various laboratories, a good practice is to report the performance of the DSSC with the dye under investigation together with that of a control cell fabricated under similar conditions with **N719** or **N3** as standard dye). This efficiency was reduced to 2.5% (η_rel_ = 28%) after the application of a black mask, confirming that, for a better comparison of the photoelectrochemical performance of DSSCs, it is important to specify whether the devices are masked or unmasked, being the photocurrents influenced by photons scattered into the TiO_2_ spot by the reflective surfaces of the cell [19].

The fact that the high level of symmetry of the homoleptic complexes could hamper an efficient directional movement of the photogenerated electrons led to the investigation of heteroleptic copper(I) complexes, with a *push-pull* structure as photosensitizers for DSSCs. In these kinds of complexes, one ligand bears an electron-withdrawing group suitable for anchoring, while the second ligand carries an electron-releasing group to provide directionality for better electron injection in the TiO_2_ conduction band (Scheme 1, **B**). One of the best efficiencies (η = 2.88%; η_rel_ = 38.1% with a masked cell) has been obtained for a heteroleptic copper(I) complex bearing a phosphonic acid anchoring ligand and 2-(6-methylpyridin-2-yl)thiazole) as an ancillary ligand (**Cu3**, Scheme 2; V_oc_ = 530 mV, FF = 0.70, J_sc_ = 7.76 mA cm^−2^) [20]. The highest efficiency (η =4.66%; η_rel_ = 63%) has been reported for a heteroleptic copper(I) complex bearing an anchoring 4,4′-dicarboxylic acid bipyridine with two mesityl groups in positions 6 and 6′, and an ancillary 4,4′-bis(styryl)-2,2′-bipyridines with NEt_2_ donor substituents (**Cu4**, Scheme 2; V_oc_ = 610 mV, FF = 0.71, J_sc_ = 10.86 mA cm^−2^) in the presence of chenodeoxycholic acid to prevent dye aggregation on the semiconductor surface [21], but it was not specified if the cell was masked or not. 

In this section, examples of homoleptic and heteroleptic copper(I) complexes investigated in the last two years as dyes in DSSCs will be given. 

A novel design of *push-pull* complexes, developed in 2023 by Housecroft and co-workers, will also be illustrated. In this strategy, a single asymmetrical ligand bears both electron-withdrawing (acceptor) and electron-donating (donor) substituents (**C**, see Scheme 1). Additionally, since cosensitization with dyes that absorb in complementary parts of the visible spectrum could be a good approach to extending the absorption of solar light, some authors investigated this strategy to improve the photoconversion efficiency of copper-based DSSCs. Here, examples, reported in the last two years, of DSSCs cosensitized with a copper(I) complex will be given.

Copper complexes designed to be applied as dyes in solar cells, and here reported, generally present chelating ligands belonging to different families, such as the N^N (phenanthrolines, bipyridines, and pyridyl-quinolines) or N^O ones (carboxyl-pyridines, hydroxy-quinolines, and salicylimine). These kinds of ligands are useful in order to guarantee stability to the obtained complexes by effectively binding the metal atom and preventing replacement by other species that may be present in the cell formulation.

In order to obtain high-performing DSSCs, the absorbance of the incident sunlight must be optimal. The more the absorption range of the dye overlaps with the spectrum of the incident sunlight, the higher the possibility of having high conversion efficiencies. A problem with copper(I) dyes is that the related metal-to-ligand charge transfer transitions typically lie between 430 and 570 nm, a range that can be broadened by increasing the π-system of the ligands. Cosensitization with dyes that absorb in complementary parts of the visible spectrum is an appealing approach to solving this problem [22,23,24,25]. Following this strategy, in 2017, Housecroft et al. [26] combined **Cu3** (Scheme 2) with a commercially available squaraine derivative, achieving the highest photoconversion efficiency reported for a masked copper-based DSSC (η = 4.51%; 65.6% relative to **N719** set at 100%). This high performance achieved by the addition of squaraine led to a large increase in J_sc_ (12.26 mA cm^−2^) but similar V_oc_ (520 mV) and FF (0.71) and showed the great potential of cosensitization for copper-based DSSCs. 

**Figure 2:**
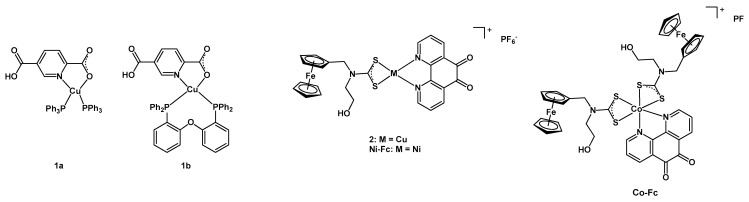
Structure of dyes **1a**, **1b**, **2**, **Ni-Fc** and **Co-Fc**.

Following the cosensitization approach, in 2022 Campos-Gaxiola and coworkers published two new Cu(I) complexes (structure in Figure 2) [27] sharing the monoanionic form of pyridine-2,5-dicarboxylic acid as N^O bidentate ligand; the two other coordination sites on the metal were occupied by two triphenylphosphines in the case of **1a** and by the chelating bis [2-(diphenylphosphine)phenyl]ether in **1b**.

The mentioned complexes were tested as cosensitizers in the preparation of DSSCs by employing an equimolar amount of the copper(I) complex and of the standard dye **N719** in the presence of the traditional I^−^/I_3_^−^ redox couple and with a platinum counterelectrode. For comparison’s sake, a solar cell having only **N719** (twice the quantity with respect to the devices presenting the sensitizers’ mixture) was also produced.

The **1a**/**N719** mixture provided a V_oc_ of 652 mV and a J_sc_ of 1.580 mA cm^−2^, with an efficiency of 2.92% (Table 1, entry 1); these values were slightly higher than those obtained by using **1b** as codye, since in that case the open-circuit voltage, the short-circuit current, and the efficiency were 643 mV, 1.446 mA cm^−2^ and 2.69%, respectively (entry 2).

Even if, in any case, the best results were the ones achieved by using dye **N719** alone, the application of the Cu(I) compounds **1a** and **1b** as cosensitizers could reach a relative efficiency of 63.6% and 58.6%, respectively, when compared to the reference cell with **N719** alone; this was a remarkable outcome, since only half the amount of the ruthenium-based dye was employed in the DSSCs with copper cosensitizers and the resulting efficiency was more than half of that of the cell containing only **N719**: 2.92% and 2.69% for **1a** and **1b**, respectively, vs. 4.59%. This work confirms the great potential of copper complexes as cosensitizers [26].

Another example of cosensitization of solar cells is represented by the work published in the same year by Chauhan and Kumar [28]; here the copper(II) center was bound to a neutral 1,10-phenanthroline-5,6-dione and to a ferrocene-bearing dithiocarbamate (**2**). Moreover, the analogous nickel(II) (**Ni-Fc**) and cobalt(III) (**Co-Fc**) complexes were synthesized; the Co(III) compound had an octahedral coordination and presented one phenanthroline and two dithiocarbamate-based ligands. Figure 2 shows the mentioned compounds.

Not only single complexes **2**, **Ni-Fc**, and **Co-Fc** were tested as dyes in the preparation of DSSCs, but also the mixtures composed by **2** + **N719**, **Co-Fc** + **N719**, and by **2** + **Co-Fc** + **N719**. The choice of the cosensitization strategy arises from the fact that the novel dyes alone cannot efficiently cover the entire absorption region, thus limiting light harvesting and consequently the device’s performance.

While the results provided by complex **2** alone were not so good, still having low current, voltage, and efficiency, the three-component mixture provided the best results, even largely overcoming those obtained by **N719** alone in the same experimental conditions, with a V_oc_ of 733 mV (vs. 718 mV of **N719**), a J_sc_ of 12.87 mA cm^−2^ (v.s 9.68 mA cm^−2^) and an efficiency of 6.05% (vs. 4.39%). Promising results were also provided by the combination of **Co-Fc** with **N719**, with a voltage of 730 mV, a photocurrent of 11.41 mA cm^−2^ and an efficiency of 5.31%. Complete data are reported in Table 1, entries 4–10.

This experiment clearly shows how the use of mixtures of dyes could be beneficial for the photovoltaic performances of solar cells, employing less expensive and much more abundant metals than Ru as cosensitizers, thus reducing the needed amount of a standard Ru-based sensitizer such as the classical **N719**.

**Figure 3:**
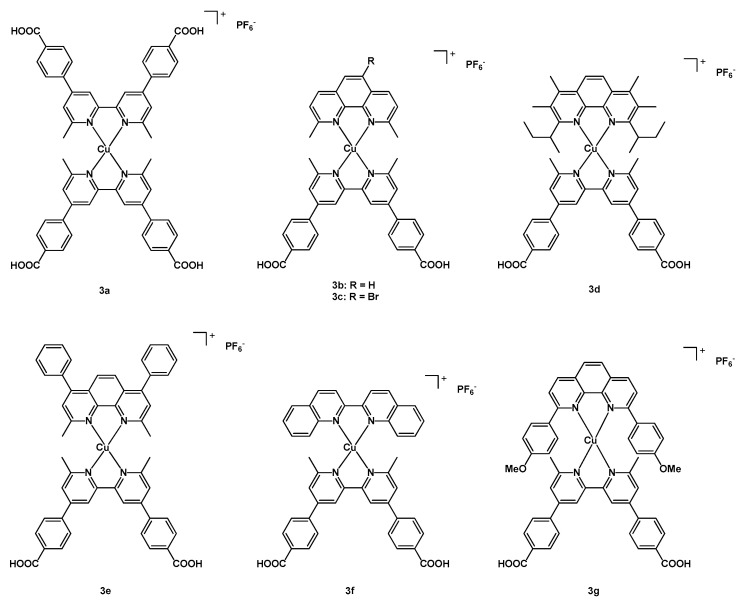
Structure of dyes **3a**–**3g**.

In 2022, Gardner et al. proposed one homoleptic Cu(I) complex and six new heterleptic Cu(I) compounds, following the traditional *push-pull* architecture presented in Scheme 1, to be tested as dyes in DSSCs [29]. The latter compounds shared the same bidentate ligand, i.e., a 6,6′-dimethyl-2,2′-bipyridine-4,4′-dibenzoic acid (dbda, whose role was to bind the molecule to the TiO_2_ surface), while the second N^N bidentate ligand was a 1,10-phenanthroline, a 2,2′-bipyridine, or a 2,2′-biquinoline (see Figure 3 for the structure of complexes **3a**–**3g**).

In particular, the authors tested the following ancillary ligands: 2,9-dimethyl-1,10-phenanthroline (dmp, in **3b**); 5-bromo-2,9-dimethyl-1,10-phenanthroline (Br-dmp, in **3c**); 2,9-di(secbutyl)-3,4,7,8-tetramethyl-1,10-phenanthroline (dsbtmp, in **3d**); 2,9-dimethyl-4,7-diphenyl-1,10-phenanthroline (bcp, in **3e**); 2,2′-biquinoline (biq, in **3f**); and 2,9-dianisyl-1,10-phenanthroline (dap, in **3g**).

After finding out that the iodine-based electrolyte was the most suitable, all the copper complexes were used to prepare solar cells; moreover, a reference cell sensitized with **N719** was assembled.

For all dyes **3a**–**3f**, the open-circuit voltage was in the range 550–566 mV, with a photocurrent between 2.87 mA cm^−2^ (for **3a**, Table 1, entry 11) and 4.79 mA cm^−2^ (in the case of **3d** and **3e**, entries 14–15); only for one sensitizer (namely **3e**, entry 15), the efficiency reached a value of 2%. Considering the photovoltaic results of the cell having compound **N719** (entry 18), they were in all cases much more relevant than the tested new complexes, with a V_oc_ of 700 mV, a J_sc_ of 17.81 mA cm^−2^, and an efficiency of 7.60%. Table 1 summarizes all the detailed results for the prepared cells. The performance of the device sensitized with the reference dye **N719** was remarkably better than that of those having the new Cu dyes, this being due to the greater light harvesting ability of the Ru complex (in agreement with the molar extinction coefficients: ~7500 M^−1^ cm^−1^ for the copper complexes and ~15,000 for **N719**) and broader absorption spectra (350–800 nm vs. 350–650 nm).

As a general consideration, the authors pointed out that the design of the ancillary ligand to be included in the heteroleptic complex together with the binding dbda is of crucial importance; in general, push-pull structures provided better results, so a more electron-donating bidentate ligand could be beneficial for the performances, together with higher molar extinction coefficients and a broader absorption region, to enhance the electron injection onto the titania surface.

**Table 1:** Photovoltaic data of solar cells produced with copper-based dyes **1a**–**1b**, **2** and **3a**–**3g** ^a^.

Entry	Dye	Redox Couple	V_oc_ (V)	J_sc_ (mA cm^−2^)	FF	η (%)	η_rel_ (%)	CE	Ref.
1	**1a** + **N719** ^b^	I^−^/I_3_^−^	0.652	1.580	0.566	2.92	63.6	Pt	[26]
2	**1b** + **N719** ^b^	I^−^/I_3_^−^	0.643	1.446	0.579	2.69	58.6	Pt	[26]
3	**N719** ^b^	I^−^/I_3_^−^	0.708	2.265	0.572	4.59	-	Pt	[26]
4	**2** ^c^	I^−^/I_3_^−^	0.616	5.78	0.64	2.27	51.7	Pt	[28]
5	**Ni-Fc** ^c^	I^−^/I_3_^−^	0.610	3.83	0.63	1.48	33.7	Pt	[28]
6	**Co-Fc** ^c^	I^−^/I_3_^−^	0.620	7.65	0.63	3.01	68.5	Pt	[28]
7	**2** + **N719** ^c^	I^−^/I_3_^−^	0.728	10.67	0.63	4.91	112	Pt	[28]
8	**Co-Fc** + **N719** ^c^	I^−^/I_3_^−^	0.730	11.41	0.64	5.31	121	Pt	[28]
9	**2** + **Co-Fc** + **N719** ^c^	I^−^/I_3_^−^	0.733	12.87	0.64	6.05	138	Pt	[28]
10	**N719** ^c^	I^−^/I_3_^−^	0.718	9.68	0.63	4.39	-	Pt	[28]
11	**3a** ^d^	I^−^/I_3_^−^	0.550	2.87	0.74	1.17	15.4	Pt	[29]
12	**3b** ^d^	I^−^/I_3_^−^	0.563	3.31	0.74	1.38	18.1	Pt	[29]
13	**3c** ^d^	I^−^/I_3_^−^	0.555	3.17	0.70	1.23	16.2	Pt	[29]
14	**3d** ^d^	I^−^/I_3_^−^	0.563	4.79	0.68	1.81	23.8	Pt	[29]
15	**3e** ^d^	I^−^/I_3_^−^	0.565	4.79	0.73	2.05	27.0	Pt	[29]
16	**3f** ^d^	I^−^/I_3_^−^	0.553	3.35	0.67	1.24	16.3	Pt	[29]
17	**3g** ^d^	I^−^/I_3_^−^	0.566	4.16	0.72	1.73	22.7	Pt	[29]
18	**N719** ^d^	I^−^/I_3_^−^	0.700	17.81	0.61	7.60	-	Pt	[29]

^a^ under AM 1.5 simulated light source; TiO_2_ employed as semiconductor; η_rel_ = efficiency relative to reference dye N719, set to 100%; CE: counterelectrode. ^b^ 0.3 mM dye in 1:1 (*v*/*v*) CH_3_CN/*t*BuOH. Electrolyte composed of 0.05 M I_2_ + 0.1 M LiI + 0.5 M TBP + 0.6 M NBu_4_I in 1:1 (*v*/*v*) CH_3_CN/3-methoxypropionitrile (TBP = 4-*t*Bu-pyridine). ^c^ dye solution in 1:1 (*v*/*v*) DMF/EtOH, concentration not specified. Electrolyte composed of 0.05 M I_2_ + 0.05 M LiI + 0.5 M TBP in CH_3_CN. ^d^ the titania substrates were soaked in a 1.0 mM MeOH solution of ligand dbda for 24 h; then each functionalized electrode was soaked for 24 h in a 1 mM CH_3_CN solution of the desired homoleptic complexes [Cu(dmp)_2_]^+^, [Cu(Br-dmp)_2_]^+^, [Cu(bcp)_2_]^+^, [Cu-(dsbtmp)_2_], and [Cu(dap)_2_]^+^, or in a CH_3_CN solution containing 1 mM [Cu(CH_3_CN)_4_]PF_6_ and 2 mM biq ligand or alternatively in a MeOH solution containing 1 mM [Cu(CH_3_CN)_4_]PF_6_ and 2 mM of the dbda ligand. Electrolyte composed of 0.65 M 1-butyl-3-methylimidazolinium iodide + 0.025 M LiI + 0.04 I_2_ + 0.28 M TBP in 85:15 (*v*/*v*) CH_3_CN/valeronitrile.

**Figure 4:**
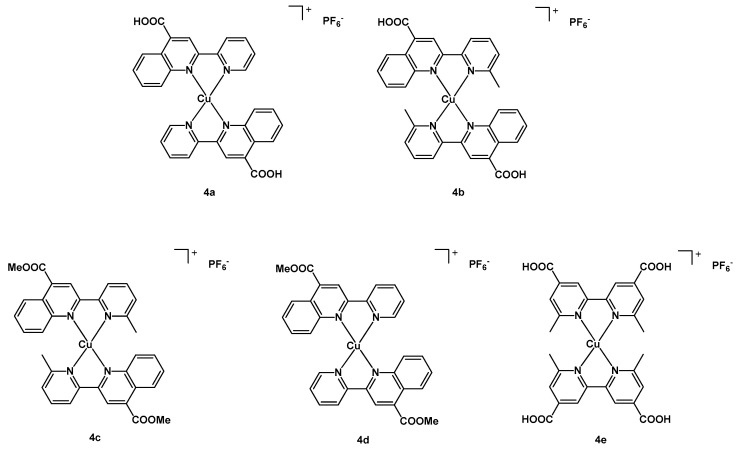
Structure of dyes **4a**–**4e**.

Falaras and Philippopoulos published, in 2022, their results concerning new homoleptic copper(I) complexes tested as dyes in solar cells [30]. Compound **4a** had a 2-(2′-pyridyl)quinoline with a carboxylic group in position 4, with the addition of a methyl group in position 6′ in the case of **4b**; complexes **4c** and **4d** had, respectively, the same structure as **4a** and **4b**, but with the carboxylic group converted into its methyl ester. Finally, in **4e**, the N^N ligand was a 6,6′-dimethyl-2,2′-bipyridine bearing COOH groups in positions 4 and 4′. Figure 4 shows the molecular structure of dyes **4a**–**4e**.

The aforementioned complexes were tested as sensitizers for the preparation of some DSSCs, employing the I^−^/I_3_^−^ couple as redox shuttles and a counterelectrode composed of platinum. The best results were achieved by compound **4b**, providing a V_oc_ of 591 mV, a J_sc_ of 2.94 mA cm^−2^ and an efficiency of 1.20% (Table 2, entry 6); in the case of complexes **4a** and **4b**, also different architectures of the semiconductor component were tested, as reported in the footnotes of Table 2.

Research on DSSCs based on coordination polymers is less developed than on mononuclear metal complexes, probably because the process of preparing thin films of coordination polymers has not been well studied yet [31].

**Figure 5:**
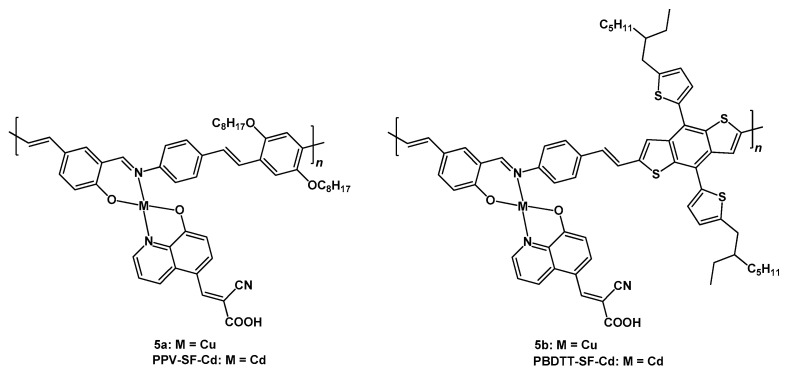
Structure of dyes **5a**, **PPV-SF-Cd**, **5b**, and **PBDTT-SF-Cd**.

This approach was followed by Zhong and coworkers in 2022 [32], involving the use of polymeric copper(II) (**5a** and **5b**) and cadmium(II) (**PPV-SF-Cd** and **PBDTT-SF-Cd**) complexes as dyes in DSSCs. The proposed compounds were characterized by a D-A-π-A structure (where D and A stand for an electron-donor or electron-accepting group, respectively, and π for a π-conjugated bridge) by adding an auxiliary electron-accepting unit in the sensitizer’s molecular structure. Figure 5 shows the structures of the discussed complexes.

In the discussed dyes, the role of the additional A-moiety was played by the metal complex, whose electron-withdrawing ability could be tuned by modifying the ligands and their substituents.

The Cu(II) and Cd(II) compounds shared the same coordination around the metal center; the first ligand was an 8-hydroxy-quinoline with a cyanoacrylic group in position 5, employed to bind the compound to the titania surface of the device; the second bidentate N^O ligand was an *N*-aryl-salicylimine bearing an aromatic moiety, this being a di-octyloxy benzene in the case of **5a** and **PPV-SF-Cd** and a substituted di-thienyl-benzo(dithiophene) in **5b** and **PBDTT-SF-Cd**.

Concerning the solar cells prepared with the described polymers, **5a** provided an open-circuit voltage of 0.69 V, a J_sc_ of 9.80 mA cm^−2^, and an efficiency of 4.77% (Table 2, entry 10); much more remarkable was the performance of the cell sensitized by testing **5b**, since it provided a V_oc_ of 0.79 V, a J_sc_ of 14.86 mA cm^−2^, and an efficiency of 8.45% (entry 11).

The performances of the cells sensitized with the cadmium-containing polymers were slightly better when compared with those of the devices based on copper (entries 12–13), this being probably due to the bigger radius of the metal cation, which could explain a greater electron-withdrawing ability of the auxiliary acceptor and charge transfer between donor and acceptor.

These data show how the accurate design of the dye, together with the possibility of using polymeric species combined with coordination compounds, could provide encouraging results for the production of solar cells based on nonexpensive metals such as copper.

**Table 2:** Photovoltaic data of solar cells produced with copper-based dyes **4a**–**4e** and **5a**–**5b** ^a^.

Entry	Dye	Redox Couple	V_oc_ (V)	J_sc_ (mA cm^−2^)	FF	η (%)	CE	Ref.
1	**4a** ^b,c^	I^−^/I_3_^−^	0.446	0.26	0.66	0.08	Pt	[30]
2	**4a** ^b,d^	I^−^/I_3_^−^	0.470	0.60	0.55	0.15	Pt	[30]
3	**4b** ^b,c^	I^−^/I_3_^−^	0.465	0.41	0.56	0.11	Pt	[30]
4	**4b** ^b,d^	I^−^/I_3_^−^	0.585	2.87	0.68	1.15	Pt	[30]
5	**4b** ^b,e^	I^−^/I_3_^−^	0.549	2.42	0.64	0.85	Pt	[30]
6	**4b** ^b,f^	I^−^/I_3_^−^	0.591	2.94	0.69	1.20	Pt	[30]
7	**4c** ^b,f^	I^−^/I_3_^−^	0.576	2.90	0.63	1.05	Pt	[30]
8	**4d** ^b,d^	I^−^/I_3_^−^	0.480	0.37	0.57	0.10	Pt	[30]
9	**4e** ^b,d^	I^−^/I_3_^−^	0.443	0.28	0.43	0.05	Pt	[30]
10	**5a** ^g^	I^−^/I_3_^−^	0.69	9.80	0.7054	4.77	Pt	[32]
11	**5b** ^g^	I^−^/I_3_^−^	0.79	14.86	0.7198	8.45	Pt	[32]
12	**PPV-SF-Cd** ^g^	I^−^/I_3_^−^	0.73	10.28	0.7086	5.30	Pt	[32]
13	**PBDTT-SF-Cd** ^g^	I^−^/I_3_^−^	0.79	14.94	0.7261	8.59	Pt	[32]

^a^ under AM 1.5 simulated light source; TiO_2_ employed as semiconductor; CE: counterelectrode. ^b^ dye solutions approximately 1.2·10^−4^ M in acetone (MeOH for **4e**). Electrolyte composed of 1 M 1,3-dimethyilimidazolium iodide + 50 mM LiI + 0.5 M TBP + 0.1 M guanidinium thiocyanate in 85:15 (*v*/*v*) CH_3_CN/butyronitrile. ^c^ on a photoanode consisting of transparent titania paste. ^d^ on a photoanode consisting of active opaque titania paste + scattering layer. ^e^ on a photoanode consisting of a 3-μm transparent titania layer + active opaque titania paste + scattering layer. ^f^ on a photoanode consisting of a compact layer + active opaque titania paste + scattering layer. ^g^ dye solution of 0.2 mM in DMF. The electrolyte is composed of 0.1 M 1,2-dimethyl-3-propylimidazolium iodide + 0.05 M LiI + 0.6 M I_2_ + 0.5 M TBP in CH_3_CN.

Additionally, an interesting aspect investigated in 2023 was the possibility of obtaining stable devices using compounds prepared from natural sources.

**Figure 6:**
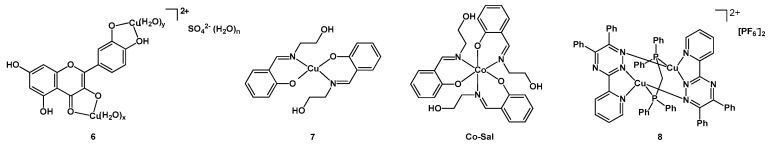
Structure of dyes **6**, **7**, **Co-Sal**, and **8**.

Thus, Özaydın and Gözel published the copper(II) complex **6** (Figure 6) derived from the reaction of CuSO_4_·5H_2_O with quercetin [33]. Quercetin (3,5,7-trihydroxy-2-(3,4-dihydroxyphenyl)-4Hchromen-4-one) is a flavonoid naturally occurring in many plants, such as tomato and onion; the presence of carbonyl and hydroxyl groups allows for its binding to metal cations to form transition metal complexes. The resulting compounds are colored and often fluorescent, so a possible application in electronic devices could be tested.

In this paper, both compound **6** and the standard Ru-based sensitizer **N3** were employed in the preparation of DSSCs, whose photovoltaic properties were recorded not only immediately after the production but also every week for five consecutive weeks.

The best performance was in any case provided by **N3**, with a starting V_oc_ of 0.76 V, a J_sc_ of 1.05 mA cm^−2^ and an efficiency of 0.199% (Table 3, entry 7); such values remained substantially stable even after the 5-week period. Concerning the Cu-quercetin complex, the starting voltage of 0.60 V remained unchanged, while it is interesting to point out that the short-circuit current and the efficiency increased during the 5 weeks, reaching values of 0.41 mA cm^−2^ and 0.082%, respectively (entry 6).

Considering the important photovoltaic difference between the Cu-quercetine dye and the standard complex **N3**, the authors claim that this is due to the fact that the devices sensitized with the copper-based compounds present a slower recombination.

Even if the reported results were not competitive with those of the cells sensitized with classical dyes based on ruthenium, this work shows how it is possible to obtain stable devices using compounds prepared from natural sources.

In the same year, Chauhan, Kumar, and coworkers published new homoleptic Cu(II) and Co(III) complexes (compounds **7** and **Co-Sal**, structure in Figure 6) having a bidentate N^O ligand obtained by the condensation of salicylaldehyde with ethanolamine [34].

Complex **7** was employed to sensitize a solar cell, providing a V_oc_ of 0.632 V, a J_sc_ of 7.84 mA cm^−2^ and an efficiency of 3.00% (Table 3, entry 7).

Better results were given by the device having **Co-Sal** as dye, since the open-circuit voltage reached 0.648 V, the short-circuit current was 9.75 mA cm^−2^ and the efficiency had a value of 3.84%. As stated by the authors, the higher photovoltaic performance of the cobalt-based complex arose from its higher ability to absorb light.

After their cosensitization work in 2022, in the following year, Campos-Gaxiola and coworkers presented a similar strategy using the new dinuclear Cu(I) complex **8** as a codye together with the well-known compound **N719** [35].

In this new complex, each copper center was bound to a chelating pdpt (3-(2-pyridyl)-5,6-diphenyl-1,2,4-triazine), to a monodentate pdpt ligand, and to a bridging dppm (dppm = bis(diphenylphosphino)methane), as shown in Figure 6.

The authors described the preparation of two DSSCs; the first having a 1:1 mixture of dyes **8** and **N719**, and the latter sensitized only with **N719** (Table 3, entries 15 and 16); the photovoltaic results were important since, by using half the amount of Ru complex, a relative yield of 92% (when compared to **N719** alone) was achieved, with a J_sc_ of 5.095 mA cm^−2^ and an efficiency of 2.03%.

**Table 3:** Photovoltaic data of solar cells produced with copper-based dyes **6**, **7**, and **8** ^a^.

Entry	Dye	Redox Couple	V_oc_ (V)	J_sc_ (mA cm^−2^)	FF	η (%)	η_rel_ (%)	CE	Ref.
1	**6** ^b,c^	I^−^/I_3_^−^	0.60	0.35	0.32	0.067	33.7	Pt	[33]
2	**6** ^b,d^	I^−^/I_3_^−^	0.58	0.40	0.32	0.074	38.4	Pt	[33]
3	**6** ^b,e^	I^−^/I_3_^−^	0.60	0.45	0.30	0.081	44.2	Pt	[33]
4	**6** ^b,f^	I^−^/I_3_^−^	0.58	0.43	0.32	0.080	42.8	Pt	[33]
5	**6** ^b,g^	I^−^/I_3_^−^	0.60	0.44	0.35	0.093	48.2	Pt	[33]
6	**6** ^b,h^	I^−^/I_3_^−^	0.58	0.41	0.35	0.082	42.0	Pt	[33]
7	**N3** ^b,c^	I^−^/I_3_^−^	0.76	1.05	0.25	0.199	-	Pt	[33]
8	**N3** ^b,d^	I^−^/I_3_^−^	0.74	1.04	0.25	0.193	-	Pt	[33]
9	**N3** ^b,e^	I^−^/I_3_^−^	0.74	0.99	0.25	0.183	-	Pt	[33]
10	**N3** ^b,f^	I^−^/I_3_^−^	0.74	1.01	0.25	0.187	-	Pt	[33]
11	**N3** ^b,g^	I^−^/I_3_^−^	0.76	1.01	0.25	0.193	-	Pt	[33]
12	**N3** ^b,h^	I^−^/I_3_^−^	0.76	1.04	0.25	0.195	-	Pt	[33]
13	**7** ^i^	I^−^/I_3_^−^	0.632	7.84	0.60	3.00	-	Pt	[34]
14	**Co-Sal** ^i^	I^−^/I_3_^−^	0.648	9.75	0.61	3.84	-	Pt	[34]
15	**8 + N719** ^j^	I^−^/I_3_^−^	0.757	5.095	0.527	2.03	92.3	Pt	[35]
16	**N719** ^j^	I^−^/I_3_^−^	0.770	6.030	0.473	2.2	-	Pt	[35]

^a^ under AM 1.5 simulated light source; TiO_2_ employed as semiconductor; η_rel_ = efficiency relative to reference dyes N3 or N719, set to 100%; CE: counterelectrode. ^b^ dye solutions 0.01 M, solvent not specified. Electrolyte formulation not specified. ^c^ measurements performed immediately after the preparation of the cell. ^d^ measurements performed after 1 week. ^e^ measurements performed after 2 weeks. ^f^ measurements performed after 3 weeks. ^g^ measurements performed after 4 weeks. ^h^ measurements performed after 5 weeks. ^i^ dye solutions in 1:1 (*v*/*v*) CH_2_Cl_2_/EtOH, concentration not specified. Electrolyte composed of 0.05 M I_2_ + 0.05 M LiI + 0.5 M TBP in CH_3_CN. ^j^ Dye solutions 0.3 mM in in 1:1 (*v*/*v*) CH_3_CN/*t*BuOH. Electrolyte composed of 0.05 M I_2_ + 0.1 M LiI + 0.5 M TBP + 0.6 M NBu_4_I in 1:1 (*v*/*v*) CH_3_CN/3-methoxypropionitrile.

In 2023, Constable et al. published a paper [36] describing new substituted 2,2′-bipyridines employed as chelating ligands for the synthesis of copper(I) complexes to be tested as dyes in DSSCs. In this case, a new approach aimed at modifying the direction of the push-pull architecture usually present in most sensitizers, *i.e.* not by having heteroleptic complexes with electron-withdrawing groups on the anchoring ligand and donor substituents on the ancillary one (see Scheme 1, compound **C**), but by testing homoleptic compounds in which the two functionalities are both present on the same chelating ligand (Scheme 1, **C**), also having an anchoring group such as a phosphonate (D-π-A-type scaffold).

**Figure 7:**
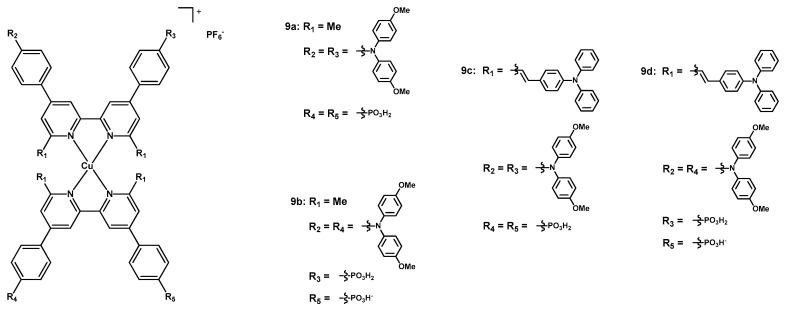
Structure of dyes **9a**–**9d**.

As it can be seen in Figure 7, in **9b** and **9d** the two pyridyl units of the ligand presented an electron-withdrawing anchoring 4-PO_3_H_2_-phenyl and a donating di(4-OMe-phenyl)amino-phenyl, respectively, while in **9a** and **9c** the pyridines of the same ligand had a symmetrical structure with the same substituents.

Moreover, for comparison’s sake, the moieties in positions 6 and 6′ (having the aim to hamper the molecular flattening upon excitation by solar light) were modified, being a simple methyl in **9a** and **9b** and a vinyl-triphenylamine in **9c** and **9d**; this different functionalization was introduced to observe the effects of further expansion of the aromatic system of the ligand.

Dyes **9a**–**9d** were tested as sensitizers in solar cells, having a Pt counterelectrode and an iodine-based electrolytic mixture; the Cu(I) complexes were deposited and anchored onto the electrodes through different techniques, as explained in detail in the paper.

As a reference, a cell sensitized with standard **N719** was also prepared. Photovoltaic data resumed in Table 4 (entries 1–5) show how the highest V_oc_ was provided by complex **9b** (599 mV), while the best short-circuit current (6.81 mA cm^−2^) and efficiency (2.54%) were provided by complex **9d**. This is an important result to confirm the usefulness of the new strategy, which has heteroleptic compounds of the [(DπA)_2_Cu]^+^ type with both “anchoring” and “ancillary” characters on the same molecule.

Even if such new dyes provide interesting features for application in DSSCs, the best-performing sensitizer was still the Ru-based **N719** (entry 5), reaching a J_sc_ > 15 mA cm^−2^ and an efficiency of 5.42%. This result was mainly due to the broader absorption region when compared to the copper(I) complexes; dyes **9c** and **9d** were able to improve the absorption by extending it thanks to the presence of the alkenyl-NPh_3_ moieties on the pyridine rings, but still not reaching the results of **N719**.

**Table 4:** Photovoltaic data of solar cells produced with copper-based dyes **9a**–**9d** ^a^.

Entry	Dye	Redox Couple	V_oc_ (V)	J_sc_ (mA cm^−2^)	FF	η (%)	η_rel_ (%)	CE	Ref.
1	**9a** ^b^	I^−^/I_3_^−^	0.531	4.90	0.68	1.78	32.8	Pt	[36]
2	**9b** ^c^	I^−^/I_3_^−^	0.599	4.77	0.64	1.82	33.6	Pt	[36]
3	**9c** ^b^	I^−^/I_3_^−^	0.503	4.90	0.71	1.76	32.5	Pt	[36]
4	**9d** ^c^	I^−^/I_3_^−^	0.564	6.81	0.66	2.54	46.9	Pt	[36]
5	**N719** ^d^	I^−^/I_3_^−^	0.615	15.02	0.59	5.42	-	Pt	[36]

^a^ under AM 1.5 simulated light source; TiO_2_ employed as semiconductor; η_rel_ = efficiency relative to reference dye N719, set to 100%; CE: counterelectrode. Electrolyte composed of 0.05 M I_2_ + 0.1 M LiI + 0.5 M 1-methylbenzimidazole + 0.6 M 1-butyl-3-methylimidazolinium iodide in 3-methoxypropionitrile. ^b^ electrodes sensitized via method c as explained in ref. [36]. ^c^ electrodes sensitized via method b as explained in ref. [36]. ^d^ dye solution 0.3 mM in EtOH.

### 2.2. Copper Complexes Employed as Redox Mediators

The redox mediator is an essential element in DSSCs since it allows for the regeneration of the fundamental state of the dye. In the last couple of decades, the I^−^/I_3_^−^ redox couple has dominated, however, despite disadvantages such as the loss of V_oc_, some complementary absorption with the sensitizer, and its volatile nature. In addition, for the I^−^/I_3_^−^ redox couple, the redox potential is almost fixed, thus choosing an appropriate photosensitizer is imperative.

Metal complexes with variable oxidation states are the best alternative as redox shuttles due to the change in their redox potentials when varying the ligands in order to suit different photosensitizers. 

Cobalt complexes as redox mediators have some drawbacks associated with slow mass transport in the electrolyte solution and large internal reorganization energy between the high-spin d^7^ and low-spin d^6^ states. In addition, cobalt can cause health hazards, such as breathing problems, and severely affect the lungs, causing pneumonia, asthma, and wheezing.

The limitations caused by the reorganization energy are minimized by using well-designed Cu(I)/(II) complexes with a distorted tetragonal geometry, thanks to a proper steric hindrance in the alfa positions. These alternative electron shuttles are of particular interest since copper is a low-cost and environmentally friendly metal [37,38,39].

Here, three very recent examples of research on solar cells containing copper-based redox mixtures are described.

**Figure 8:**
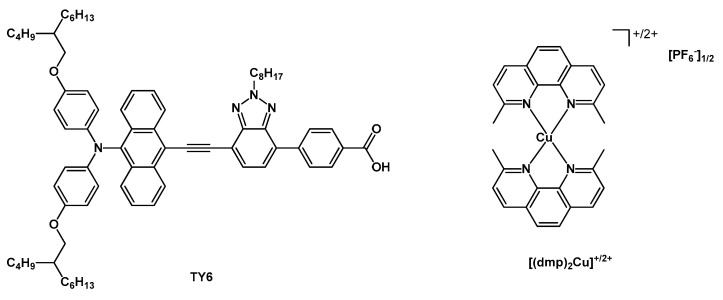
Structure of dye **TY6** and of the redox couple [(dmp)_2_Cu]^+/2+^.

In the paper published in 2023 by Yeh, Wei, and coworkers [40], the authors explored different kinds of CE to find the most suitable to couple with the copper-containing redox mixture represented by complexes [Cu(dmp)_2_]^+/2+^, where dmp is a 2,9-dimethyl-1,10-phenanthroline (Figure 8). In fact, it is important to point out that the proper selection of the CE could bring about remarkable effect on the efficiency of the device, so the same system is not always suitable for every kind of redox shuttles. As a sensitizer, the authors employed the organic compound **TY6** (structure in Figure 8).

The counterelectrodes tested by the authors presented various compositions as a consequence of the different preparation techniques, resulting in different loading and distibution of platinum onto the FTO surface: the lowest and highest amounts of the metal were obtained in the case of TR-Pt_1 and TR-Pt_10 (0.98 and 9.81 μg cm^−2^, respectively), but there the metal was randomly distributed in between the FTO grains, thus limiting its effectiveness. Better situations were those with PVA or PVO polymers, since no excessive aggregation was observed on the conductive glass.

Considering the photovoltaic performances, all CE showed similar V_oc_, ranging from 1.07 to 1.09 V, and a similar J_sc_ of ~10.4 mA cm^−2^; the best efficiencies were achieved by PVA-Pt (8.47%) and PVP-Pt (8.32%), confirming the usefulness of employing the discussed polymers in the preparation of the counterelectrodes. Table 4 summarizes the data concerning the presented devices.

In conclusion, PVA-Pt offered the best performances, not only because of the high efficiency but also because of the very low platinum amount (1.07 μg cm^−2^) needed.

**Figure 9:**
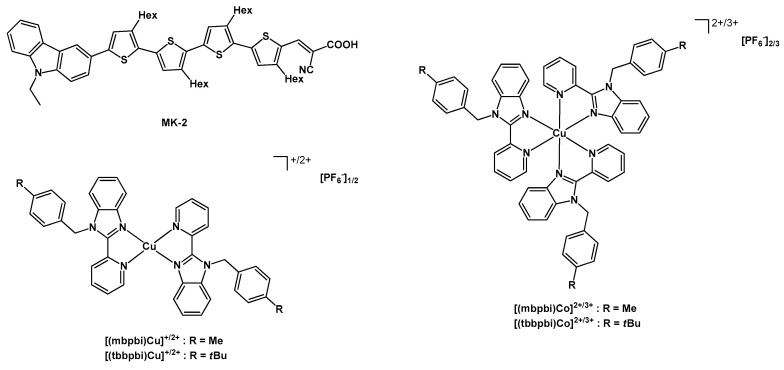
Structure of dye **MK-2** and of redox couples [(mbpbi)_2_Cu]^+/2+^, [(tbbpbi)_2_Cu]^+/2+^, [(mbpbi)_3_Co]^2+/3+^ and [(tbbpbi)_3_Co]^2+/3+^.

A different approach was followed by Shanmugam et al. in 2022 [41], with the realization of an aqueous DSSC with the organic compound **MK-2** as a sensitizer, and copper and cobalt complexes as redox mediators (see Figure 9 for the structure of both dye and redox mediators).

The Cu- and Co-based complexes shared the same bidentate chelating ligand, namely a benzyl-substituted pyridyl-benzimidazole, with different alkylic substituents in *para* position on the phenyl ring: a methyl in the case of couples [(mbpbi)_2_Cu]^+/2+^ and [(mbpbi)_3_Co]^2+/3+^, while a *tert-*butyl was present in [(tbbpbi)_2_Cu]^+/2+^ and [(tbbpbi)_3_Co]^2+/3+^.

The solar cells in which the mentioned complexes were applied were aqueous-based, thanks to the use of xanthan gum gel to disperse the electrolytic couples.

By comparing the photovoltaic performances of the prepared cells, it turned out that the best redox mediators were the Cu(I/II) ones, with [(mbpbi)_2_Cu]^+/2+^ reaching a voltage of 0.74 V, a J_sc_ of 11.02 mA cm^−2^ and an efficiency of 4.08%, and [(tbbpbi)_2_Cu]^+/2+^ presenting values of 0.73 V, 8.98 mA cm^−2^ and 3.04%, respectively (Table 4, entries 6–7). These results were remarkably higher than those provided by the cobalt-containing electrolytes (as reported in entries 8–9) because of the lower inner sphere reorganization energy typical of the discussed copper-based compounds.

The authors found out that the presence of the *t*Bu substituents instead of the simple methyl group on the benzyl moiety was beneficial for the photovoltaic data since it allowed for reduced recombination and lower reorganization energy; moreover, these complexes showed higher power conversion efficiency due to an increased redox potential (0.80 V vs. 0.60 V in the case of the Cu complexes).

**Figure 10:**
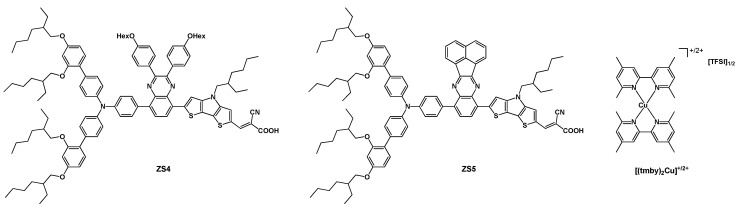
Structure of dyes **ZS4** and **ZS5** and of the redox couple [(tmby)_2_Cu]^+/2+^.

The best results achieved for a solar cell presenting a copper-based redox couple and a single sensitizer were published in 2022 by Shen, Grätzel, and coworkers [42]. In this work, the redox mediators were the [(tmby)_2_Cu]^+/2+^ couple (where tmby = 2,2′,4,4′-tetramethyl-1,1′-bipyridine), tested together with the new dyes **ZS4** and **ZS5** (dyes and redox couple are reported in Figure 10).

The new sensitizers were designed by considering the moieties that could lead to better photovoltaic performances of the final products, i.e. a dithieno[3,2-b:2″,3″-d]pyrrole as a linker and a quinoxaline as an additional electron-withdrawing unit in between the anchoring cyanoacrylic group and the triphenylamine moiety bearing ethyl-hexyloxy chains to prevent aggregation on the titania surface. The difference in the structure of the two compounds was in the substituents on the quinoxaline unit: two 4-OHex-phenyl rings in **ZS4**, while a fused naphthalene was present in **ZS5**.

As a result of such a design, very remarkable performances were achieved, in particular in the case of **ZS4**: a J_sc_ of 16.3 mA cm^−2^, a voltage of 1.05 V, and an efficiency of 13.2% (Table 5, entry 10).

Moreover, as a further positive aspect, the solar cell sensitized with the discussed dye retained 95% of its initial efficiency under continuous light soaking at 45 °C for 1000 h.

**Table 5:** Photovoltaic data of solar cells produced with copper-based redox mediators [(dmp)_2_Cu]^+/2+^, [(mbpbi)_2_Cu]^+/2+^, [(tbbpbi)_2_Cu]^+/2+^, [(mbpbi)_3_Co]^2+/3+^, [(mbpbi)_3_Co]^2+/3+^, and [(tmby)_2_Cu]^+/2+ a^.

Entry	Dye	Redox Couple	V_oc_ (V)	J_sc_ (mA cm^−2^)	FF	η (%)	CE	Ref.
1	**TY6** ^b^	[(dmp)_2_Cu]^+/2+^	1.08	9.8	0.67	7.06	TR-Pt_1 ^c^	[40]
2	**TY6** ^b^	[(dmp)_2_Cu]^+/2+^	1.08	10.4	0.70	7.84	TR-Pt_10 ^c^	[40]
3	**TY6** ^b^	[(dmp)_2_Cu]^+/2+^	1.09	10.4	0.72	8.17	PVP-Pt ^c^	[40]
4	**TY6** ^b^	[(dmp)_2_Cu]^+/2+^	1.09	10.4	0.73	8.26	PVA-Pt ^c^	[40]
5	**TY6** ^b^	[(dmp)_2_Cu]^+/2+^	1.07	10.1	0.73	7.95	PEDOT ^c^	[40]
6	**MK-2** ^d^	[(tbbpbi)_2_Cu]^+/2+ e^	0.73	8.98	0.46	3.04	Pt	[41]
7	**MK-2** ^d^	[(mbpbi)_2_Cu]^+/2+ e^	0.74	11.02	0.50	4.08	Pt	[41]
8	**MK-2** ^d^	[(tbbpbi)_3_Co]^2+/3+ f^	0.73	1.83	0.46	0.61	Pt	[41]
9	**MK-2** ^d^	[(mbpbi)_3_Co]^2+/3+ f^	0.78	3.92	0.53	1.62	Pt	[41]
10	**ZS4** ^g^	[(tmby)_2_Cu]^+/2+^	1.05	16.3	0.771	13.2	PEDOT	[42]
11	**ZS5** ^g^	[(tmby)_2_Cu]^+/2+^	0.95	14.7	0.750	10.5	PEDOT	[42]

^a^ under AM 1.5 simulated light source; TiO_2_ employed as a semiconductor; CE: counterelectrode. ^b^ 0.15 mM dye in 1:1 (*v*/*v*) *t*BuOH/CH_3_CN, with 0.3 mM chenodeoxycholic acid. Electrolyte composed of 0.2 M [(dmp)_2_Cu]TFSI + [(dmp)_2_Cu](TFSI)_2_ + 0.1 M LiTFSI + 0.6 M TBP in CH_3_CN (TFSI = trifluoromethansulfonimide). ^c^ the procedure followed to obtain the various Pt-based CE is described in ref. [40]. ^d^ 0.04 M dye in toluene. ^e^ electrolytes prepared by stirring an aqueous xanthan gum (3% weight) with a solution containing 0.2 M [(tbbpbi)_2_Cu]^+^ + 0.02 M [(tbbpbi)_2_Cu]^2+^ + 0.5 M TBP + 0.1 M LiClO_4_ in DMF. ^f^ electrolytes prepared by stirring an aqueous xanthan gum (3% weight) with a solution containing 0.2 M [(tbbpbi)_3_Co]^2+^ + 0.02 M [(tbbpbi)_3_Co]^3+^ + 0.5 M TBP + 0.1 M LiClO_4_ in DMF. ^g^ 0.06 mM dye in 3:7 (*v*/*v*) CHCl_3_/EtOH, with 1.5 mM chenodeoxycholic acid. Electrolyte composed of 0.2 M [(tmby)_2_Cu]TFSI + 0.09 M [(tmby)_2_Cu](TFSI)_2_ + 0.1 M NaTFSI + 0.6 M *N*-methylbenzimidazole in CH_3_CN.

## 3. Conclusions

Through this review, we have shown the important developments in the last two years regarding copper complexes as molecular materials for dye-sensitized solar cells. The discussed works not only confirm the great potential of well-designed copper complexes as efficient dyes, but they also provide evidence, for the first time since the pioneering work of Housecroft and co-workers in 2017, that the cosensitization strategy is a fruitful way of enhancing the photoconversion efficiency of copper-based DSSCs without the need for too complicated organic structures.

In particular, it turned out that copper complexes can be used as cosensitizers in the presence of **N719**, allowing for better photovoltaic performances with a small amount of **N719**; this cosensitization with copper, a much less expensive and much more abundant metal when compared to ruthenium, is beneficial not only for the photovoltaic performances but also for the cost of solar cells. The work conducted in the last two years confirmed that the design of novel push-pull heteroleptic complexes is more suitable than the “traditional” homoleptic complexes, bearing only electron-withdrawing groups, in order to reach better efficiencies.

However, in 2023, a novel strategy for efficient sensitizers was born: the use of homoleptic complexes of the [(DπA)_2_Cu]^+^ type, with both electron-withdrawing and electron-donating moieties on the same ligand. This novel route for copper(I) dyes is very appealing and is sure to be developed in the future.

Additionally, the review confirmed the potential of copper complexes as electron shuttles for iodine-free electrolytes, putting into evidence the importance of the nature of the counterelectrodes. Remarkably, a DSSC sensitized with a well-designed organic compound (**ZS4**) achieves an efficiency of 13.2% under AM1.5G sunlight and keeps 95% of its initial efficiency under continuous light soaking for 1000 h. This represents a record efficiency for copper-electrolyte-based DSSCs with a single sensitizer; it highlights the importance of molecular engineering of photosensitizers toward highly efficient DSSCs with copper electron shuttles.

To conclude, in the future, copper complexes are expected to play an increasingly important role both as dyes and electron shuttles in the fabrication of low-cost and stable dye-sensitized solar cells. Further studies are necessary to optimize the photovoltaic results, taking into account not only the proper molecular design of the dye but also the use of cosensitizers and the suitable choice of the electrolyte mixture, of the additives, and of the counterelectrode.

## Data Availability

No new data were created or analyzed in this study. Data sharing is not applicable to this article.

## References

[1] O’Regan B., Grätzel M. (1991). A Low-Cost, High-Efficiency Solar Cell Based on Dye-Sensitized Colloidal TiO_2_ Films. Nature.

[2] Yahya M., Bouziani A., Ocak C., Seferoğlu Z., Sillanpää M. (2021). Organic/metal-organic photosensitizers for dye-sensitized solar cells (DSSC): Recent developments, new trends, and future perceptions. Dyes Pigments.

[3] Dragonetti C., Colombo A., Magni M., Mussini P.R., Nisic F., Roberto D.M., Ugo R., Valore A., Valsecchi A., Salvatori P. (2013). Thiocyanate-Free Ruthenium(II) Sensitizer with a Pyrid-2-yltetrazolate Ligand for Dye-Sensitized Solar Cells. Inorg. Chem..

[4] Fiorini V., Marchini E., Averardi M., Giorgini L., Muzzioli S., Dellai A., Argazzi R., Sanson A., Sangiorgi N., Caramori S. (2020). New examples of Ru(II)-tetrazolato complexes as thiocyanate-free sensitizers for dye-sensitized solar cells. Dalton Trans..

[5] Mauri L., Colombo A., Dragonetti C., Roberto D., Fagnani F. (2021). Recent Investigations on Thiocyanate-Free Ruthenium(II) 2,2′-Bipyridyl Complexes for Dye-Sensitized Solar Cells. Molecules.

[6] Mathew S., Yella A., Gao P., Humphry-Baker R., Curchod B.F.E., Ashari-Astani N., Tavernelli I., Rothlisberger U., Nazeeruddin M.K., Grätzel M. (2014). Dye-sensitized solar cells with 13% efficiency achieved through the molecular engineering of porphyrin sensitizers. Nat. Chem..

[7] Kakiage K., Aoyama Y., Yano T., Oya K., Fujisawa J., Hanaya M. (2015). Highly-efficient dye-sensitized solar cells with collaborative sensitization by silyl-anchor and carboxy-anchor dyes. Chem. Commun..

[8] Zhang L., Yang X., Wang W., Gurzadyan G.G., Li J., Li X., An J., Yu Z., Wang H., Hagfeldt A. (2019). 13.6% Efficient Organic Dye-Sensitized Solar Cells by Minimizing Energy Losses of the Excited State. ACS Energy Lett..

[9] Ren Y., Zhang D., Suo J., Cao Y., Eickemeyer F.T., Vlachopoulos N., Zakeeruddin S.M., Hagfeldt A., Grätzel M. (2023). Hydroxamic acid pre-adsorption raises the efficiency of cosensitized solar cells. Nature.

[10] Colombo A., Dragonetti C., Magni M., Roberto D., Demartin F., Caramori S., Bignozzi C.A. (2014). Efficient Copper Mediators Based on Bulky Asymmetric Phenanthrolines for DSSCs. ACS Appl. Mater. Interfaces.

[11] Freitag M., Giordano F., Yang W., Pazoki M., Hao Y., Zietz B., Grätzel M., Hagfeldt A., Boschloo G. (2016). Copper Phenanthroline as a Fast and High-Performance Redox Mediator for Dye-Sensitized Solar Cells. J. Phys. Chem. C.

[12] Housecroft C.E., Constable E.C. (2015). The emergence of copper(I)-based dye sensitized solar cells. Chem. Soc. Rev..

[13] Conradie J. (2022). Polypyridyl copper complexes as dye sensitizer and redox mediator for dye-sensitized solar cells. Electrochem. Commun..

[14] Housecroft C.E., Constable E.C. (2022). Solar Energy Conversion Using First Row d-Block Metal Coordination Compound Sensitizers and Redox Mediators. Chem. Sci..

[15] Mauri L., Colombo A., Dragonetti C., Fagnani F. (2022). A Fascinating Trip into Iron and Copper Dyes for DSSCs. Inorganics.

[16] Colombo A., Dragonetti C., Roberto D., Fagnani F. (2021). Copper Complexes as Alternative Redox Mediators in Dye-Sensitized Solar Cells. Molecules.

[17] Muñoz-García A.B., Benesperi I., Boschloo G., Concepcion J.J., Delcamp J.H., Gibson E.A., Meyer G.J., Pavone M., Pettersson H., Hagfeldt A. (2021). Dye-sensitized solar cells strike back. Chem. Soc. Rev..

[18] Alonso Vante N., Nierengarten J.-F., Sauvage J.-P. (1994). Spectral Sensitization of Large-band-gap Semiconductors (Thin Films and Ceramics) by a Carboxylated Bis(1,I 0-Phenanthroline)copper(l) Complex. J Chem Soc. Dalton Trans..

[19] Colombo A., Dragonetti C., Roberto D., Valore A., Biagini P., Melchiorre F. (2013). A simple copper(I) complex and its application in efficient dye sensitized solar cells. Inorg. Chim. Acta.

[20] Malzner F.J., Prescimone A., Constable E.C., Housecroft C.E., Willgert M. (2017). Exploring simple ancillary ligands in copper-based dye-sensitized solar cells: Effects of a heteroatom switch and of co-sensitization. J. Mater. Chem. A.

[21] Sandroni M., Favereau L., Planchat A., Akdas-Kilig H., Szuwarski N., Pellegrin Y., Blart E., Le Bozec H., Boujtita M., Odobel F. (2014). Heteroleptic copper(I)–polypyridine complexes as efficient sensitizers for dye sensitized solar cells. J. Mater. Chem. A.

[22] Babu D.D., Elsherbiny D., Cheema H., El-Shafei A., Vasudeva Adhikari A. (2016). Highly efficient panchromatic dye-sensitized solar cells: Synergistic interaction of ruthenium sensitizer with novel co-sensitizers carrying different acceptor units. Dyes Pigments.

[23] Chang S., Wang H., Tien Lin Lee L., Zheng S., Li Q., Wong K.Y., Wong W.-K., Zhu X., Wong W.-Y., Xiao X. (2014). Panchromatic light harvesting by N719 with a porphyrin molecule for high-performance dye-sensitized solar cells. J. Mater. Chem. C.

[24] Yum J.-H., Jang S.-R., Walter P., Geiger T., Nüesch F., Kim S., Ko J., Grätzel M., Nazeeruddin M.K. (2007). Efficient co-sensitization of nanocrystalline TiO_2_ films by organic sensitizers. Chem. Commun..

[25] Zhao Y., Lu F., Zhang J., Dong Y., Zhang B., Feng Y. (2017). Stepwise co-sensitization of two metal-based sensitizers: Probing their competitive adsorption for improving the photovoltaic performance of dye-sensitized solar cells. RSC Adv..

[26] Malzner F.J., Willgert M., Constable E.C., Housecroft C.E. (2017). The way to panchromatic copper(I)-based dye-sensitized solar cells: Co-sensitization with the organic dye SQ2. J. Mater. Chem. A.

[27] Soto-Acosta S., Campos-Gaxiola J.J., Reynoso-Soto E.A., Cruz-Enríquez A., Baldenebro-López J., Höpfl H., García J.J., Flores-Álamo M., Miranda-Soto V., Glossman-Mitnik D. (2022). Synthesis, Crystal Structure, DFT Studies and Optical/Electrochemical Properties of Two Novel Heteroleptic Copper(I) Complexes and Application in DSSC. Crystals.

[28] Singh A., Srivastava D., Gosavi S.W., Chauhan R., Ashokkumar M., Albalwi A.N., Muddassir M., Kumar A. (2022). A double co-sensitization strategy using heteroleptic transition metal ferrocenyl dithiocarbamate phenanthrolene-dione for enhancing the performance of N719-based DSSCs. RSC Adv..

[29] Franchi D., Leandri V., Pizzichetti A.R.P., Xu B., Hao Y., Zhang W., Sloboda T., Svanström S., Cappel U.B., Kloo L. (2022). Effect of the Ancillary Ligand on the Performance of Heteroleptic Cu(I) Diimine Complexes as Dyes in Dye-Sensitized Solar Cells. ACS Appl. Energy Mater..

[30] Peppas A., Sokalis D., Perganti D., Schnakenburg G., Falaras P., Philippopoulos A.I. (2022). Sterically demanding pyridine-quinoline anchoring ligands as building blocks for copper(I)-based dye-sensitized solar cell (DSSC) complexes. Dalton Trans..

[31] Okubo T., Tanaka N., Anma H., Kim K.H., Maekawa M., Kuroda-Sowa T. (2012). Dye-sensitized Solar Cells with New One-Dimensional Halide-Bridged Cu(I)–Ni(II) Heterometal Coordination Polymers Containing Hexamethylene Dithiocarbamate Ligand. Polymers.

[32] Tian Y., Wang K., Zhang H., Wu X., Zhong C. (2022). Novel polymeric metal complexes of salicylaldehyde schiff base derivative being used for dye sensitizer. Tetrahedron.

[33] Özaydın C., Gözel M. (2023). The Use of Copper-Quercetin Complex as Photosensitizer in Dye Sensitive Solar Cells and Its Photovoltaic Performance. Braz. J. Phys..

[34] Gautam C., Srivastava D., Kociok-Köhn G., Gosavi S.W., Sharma V.K., Chauhan R., Late D.J., Kumar A., Muddassir M. (2023). Copper(II) and cobalt(III) Schiff base complexes with hydroxy anchors as sensitizers in dye-sensitized solar cells (DSSCs). RSC Adv..

[35] Peñuelas C.A., Campos-Gaxiola J.J., Soto-Rojo R., Cruz-Enríquez A., Reynoso-Soto E.A., Miranda-Soto V., García J.J., Flores-Álamo M., Baldenebro-López J., Glossman-Mitnik D. (2023). Synthesis of a New Dinuclear Cu(I) Complex with a Triazine Ligand and Diphenylphosphine Methane: X-ray Structure, Optical Properties, DFT Calculations, and Application in DSSCs. Inorganics.

[36] Risi G., Devereux M., Prescimone A., Housecroft C.E., Constable E.C. (2023). Back to the future: Asymmetrical DπA 2,2′-bipyridine ligands for homoleptic copper(I)-based dyes in dye-sensitised solar cells. RSC Adv..

[37] Kavana L., Saygili Y., Freitag M., Zakeeruddin S.M., Hagfeldt A., Grätzel M. (2017). Electrochemical Properties of Cu(II/I)-Based Redox Mediators for Dye-Sensitized Solar Cells. Electrochim. Acta.

[38] Srivishnu K.S., Prasanthkumar S., Giribabu L. (2021). Cu(II/I) redox couples: Potential alternatives to traditional electrolytes for dye-sensitized solar cells. Mater. Adv..

[39] Freitag M., Teuscher J., Saygili Y., Zhang X., Giordano F., Liska P., Hua J., Zakeeruddin S.M., Moser J.-E., Grätzel M. (2017). Dye-sensitized solar cells for efficient power generation under ambient lighting. Nat. Photonics.

[40] Nguyen V.S., Su T.S., Chen C.-C., Yeh C.-Y., Wei T.-C. (2023). Efficient counter electrode for copper (I)(II)-mediated dye-sensitized solar cells based on polyvinyl alcohol capped platinum nanoclusters. J. Taiwan Inst. Chem. Eng..

[41] Selvaraj B., Shanmugam G., Kamaraj S., Thirugnanasambandam E., Peters S., Gunasekeran A., Sambandam A., Pillai R.S. (2022). Effect of Copper and Cobalt Metal Complex Redox Mediator Based Xanthan Gum Gel Electrolyte Materials on Performance of Dye Sensitized Solar Cells. ChemistrySelect.

[42] Grobelny A., Shen Z., Eickemeyer F.T., Antariksa N.F., Zapotoczny S., Zakeeruddin S.M., Grätzel M. (2023). A Molecularly Tailored Photosensitizer with an Efficiency of 13.2% for Dye-Sensitized Solar Cells. Adv. Mater..

